# A Cloud Infrastructure for Large Scale Health Monitoring in Older Adult Care Facilities

**DOI:** 10.1093/geroni/igab046.3653

**Published:** 2021-12-17

**Authors:** Yeonsik Noh, Uchechukwu David

**Affiliations:** University of Massachusetts, Amherst, Massachusetts, United States

## Abstract

Technology development in the sub-field of older adult care has always been on the back-burner compared to other healthcare areas. But with increasing life expectancy, this is poised to change. With the increasing older adult population, the current older adult care facilities and personnel are struggling to keep up with demand. To address this matter, we proposed a distributed system infrastructure that will enable large-scale monitoring of vital signals and early detection of emergency situations in nursing homes and assisted living communities in this study. The system is mainly comprised of node devices and cloud infrastructure. The node device collects data from multiple users and forwards this data to the cloud service. Communication between the vital-sensing devices on the users and the node device is accomplished using Bluetooth Low-Energy (BLE). The Node device is implemented on a Raspberry Pi Model 3b+ which has built-in BLE capability. A cloud server consists of back-end and front-end components. The back-end handles all the data processing and logical decisions that drive the front-end. The front-end is the interface provided to the end-users, which can monitor all patients in nursing homes at the same time. We examined our system in terms of scalability, real-time operation, cost, and usability and then found that our system provides not only a smart remote monitoring solution that can provide a better aging experience for older adults and their families, but also increase automation in nursing homes leading to a reduction in running cost and an increase in capacity.

